# The effect of political turnover on corporate ESG performance: Evidence from China

**DOI:** 10.1371/journal.pone.0288789

**Published:** 2023-07-24

**Authors:** Chao Yang, Wenhan Hao, Di Song

**Affiliations:** 1 School of Accountancy, Beijing Wuzi University, Beijing, China; 2 School of Sports Business, Beijing Sport University, Beijing, China; 3 Business School, China University of Political Science and Law, Beijing, China; The University of Sydney, AUSTRALIA

## Abstract

This paper aims to investigate the effect of political turnover on corporate ESG performance in China. By analyzing data from Chinese A-share-listed companies between 2010 and 2020, we have discovered that changes in the municipal party committee secretary or the mayor of the prefecture-level city where a firm is located have a detrimental effect on corporate ESG performance. Compared with the change of the party committee, the change of mayor has a more pronounced negative impact on ESG performance. The reason behind this negative effect is primarily attributed to policy uncertainty, which leads to a decrease in governmental subsidies and an increase in ineffective under-investment by companies, consequently resulting in decreased corporate ESG performance. Furthermore, we have also observed that the adverse influence of political turnover on corporate ESG performance is relatively mitigated in SOEs, politically connected firms, and tertiary industries. These findings contribute to a deeper understanding of the relationship between political uncertainty and corporate behavior, particularly in emerging markets.

## Introduction

The study of political turnover has received growing attention as a measure of political uncertainty. Extensive research has explored its impacts on both macro and microeconomic factors. At the macro level, political turnover has been revealed to affect fiscal redistribution [1] and economic growth [2]. At the micro level, political turnover has been demonstrated to influence firms’ decision-making [3], strategic change [4], investment efficiency [5, 6], and R&D investment [7]. While previous research has predominantly focused on the impact of political turnover on factors related to economic development, it is vital to acknowledge that evaluating officials’ performance should extend beyond China’s stage of high-quality development and consider areas such as culture, society, and ecological civilization.

Recent studies have indicated that political turnover can prompt local officials to prioritize economic benefits at the expense of air quality and pollution control [8, 9]. Given that listed companies play a pivotal role in local economic development, changes in government officials profoundly impact their investments in environmental protection, social responsibility, and governance. Thus, it is imperative to investigate the influence of local official turnover on corporate ESG (Environment, Social Responsibility, and Governance) performance. ESG represents an investment philosophy and corporate rating standard that prioritizes environmental sustainability, social responsibility, and strong corporate governance over traditional financial performance. This research endeavour aims to provide insights into incentivizing firms to prioritize ESG factors in their decision-making processes, ultimately contributing to sustainable economic development.

The study scenario chosen for this research is China, motivated by two primary factors. Firstly, the appointment of local officials by higher-level communist party officials in China, rather than through local elections, introduces a level of unpredictability in the business environment for market participants [10]. Furthermore, local governments in China wield a greater degree of power compared to their counterparts in federal countries worldwide, particularly in matters of local economic affairs, environmental policies, social welfare, and other relevant aspects [11, 12]. Consequently, changes in local officials can exert significant impacts on local development. Secondly, China’s political system exhibits a prevalent issue known as "new officials ignore the old commitment," wherein incoming governments may disregard contracts, commitments, debts, and other obligations made by previous administrations and leaders, resulting in disruptions to local government policies. This policy uncertainty arising from the alteration of policies by new politicians can influence firms’ decision-making processes. In summary, the selection of China as the research setting is grounded in the country’s distinctive political and economic landscape. The appointment of local officials by higher-level communist party officials, along with the considerable power vested in local governments, fosters an unpredictable business environment for market participants. Furthermore, the challenge of "new officials ignore the old commitment" within China’s political system further complicates the continuity of local government policies, leading to policy uncertainty and subsequent effects on firms.

This study investigates the impact of local official turnover on corporate Environmental, Social, and Governance (ESG) performance in China, utilizing a sample of Chinese A-share-listed firms over the period from 2010 to 2020. The findings reveal a detrimental impact of the turnover of the municipal party committee secretary or the mayor of the prefecture-level city where the firm operates on corporate ESG performance. To delve into the distinct roles of mayors and party committee secretaries, we conduct separate tests to examine their individual effects on ESG performance. The results demonstrate that the change of mayor exerts a more pronounced negative effect on ESG performance compared to the change of the party committee. This divergence can be attributed to the division of responsibilities within the Chinese political system, as mayors bear greater responsibility for local economic matters, while party committee secretaries primarily handle officials’ appointments. Furthermore, this study delves into the underlying mechanisms through which local official turnover influences corporate ESG performance. It reveals that policy uncertainty resulting from political turnover diminishes government subsidies and promotes ineffective under-investment by companies, leading to a decline in corporate ESG performance.

To ensure the robustness and reliability of our findings, we conducted a comprehensive series of robustness tests. Firstly, we employed an alternative dependent variable, specifically the difference between the ESG score in year t and year t-1, to capture and measure the change in corporate ESG performance. This approach allowed us to validate the consistency of our results. Secondly, we employed an alternative ESG rating dataset, namely Hexun ESG, to cross-validate our regression results and assess their consistency with the baseline findings. Thirdly, recognizing that ESG comprises environmental, social, and governance scores, we conducted a granular examination of each individual score indicator. This approach enabled us to gain deeper insights into the specific effects of political turnover on each dimension of corporate ESG performance. Moreover, we introduced a control variable for the provincial governor or provincial party secretary in our regression analysis. By including this control, we accounted for their potential influence on corporate ESG performance. Encouragingly, in line with our hypothesis, all the results consistently supported our findings. Additionally, we employed an instrumental variable (IV) approach in our analysis to further address the potential endogeneity concerns, which further strengthened the robustness of our results. Moreover, to test the validity of our results and to rule out the possibility of other factors affecting corporate ESG performance, we conduct a placebo test. Specifically, we simulated a counterfactual scenario by advancing the timing of political turnover by two years, where political turnover did not occur in the actual year. The results of this placebo test revealed that all the estimated coefficients were statistically insignificant. This finding provides strong evidence that the impact of political turnover is not random and further corroborates the robustness of our results.

Through our cross-sectional analysis, several key findings have emerged. Firstly, we observed that the impact of government official turnover on corporate ESG performance differs between state-owned enterprises (SOEs) and non-state-owned enterprises. This discrepancy arises from the divergent actual controllers and interest orientations of these two types of entities. As a result, the change of government officials has a comparatively lesser impact on the ESG performance of SOEs. Secondly, we examined the role of political connections between companies and officials. We discovered that firms with stronger political connections experience reduced impact from changes in government officials and exhibit a more consistent and sustainable investment in ESG practices. This highlights the importance of political networks in mitigating the negative effects of political turnover on corporate ESG performance. Furthermore, we explored the influence of industry composition on the relationship between political turnover and ESG performance. Specifically, we found that regions with well-developed tertiary industries enjoy certain advantages in terms of economic development. Consequently, the negative impact of political turnover on corporate ESG performance is less pronounced in these tertiary industries. By uncovering these insights, our research contributes to a better understanding of how different factors shape the relationship between political turnover and corporate ESG performance.

Our study makes several significant contributions to the existing literature. Firstly, while previous research has predominantly focused on the impact of political turnover on economic development factors such as GDP [1–7], limited attention has been given to exploring the effects on areas such as culture, society, and ecological civilization. The findings of our study provide compelling evidence that the turnover of government officials can have a significant negative impact on corporate ESG performance. This adverse effect is primarily attributed to the inherent policy uncertainty resulting from political turnover and the continued reliance on GDP as the primary metric for evaluating officials’ performance. By shedding light on these dynamics, our research contributes to a deeper understanding of the challenges faced by listed companies in maintaining and improving their ESG performance in the context of political transitions.

Secondly, our study contributes to the existing literature by addressing an important aspect of ESG performance improvement that has received limited attention in previous research. ESG performance has become a pivotal indicator utilized by investors in their stock selection process [13–16]. However, the impact of political turnover on ESG performance has remained relatively unexplored. Our study seeks to fill this gap by investigating the influence of political turnover on ESG performance, providing valuable insights into how companies can enhance their ESG performance in the face of political transitions. By examining this previously unexplored relationship, our research contributes to a more comprehensive understanding of the factors that can drive improvements in ESG performance.

Thirdly, our study identifies two crucial mechanisms through which political turnover influences corporate ESG performance. We observed a decrease in governmental subsidies, which consequently hampers companies’ ability to invest effectively in ESG initiatives. Political turnover leads to an increase in companies’ ineffective under-investment, further exacerbating the decline in corporate ESG performance. By unraveling these transmission channels, our findings underscore the importance of stable political environments and policy continuity in fostering sustainable business practices within the Chinese context.

The remainder of this paper proceeds as follows. Section 2 introduces the literature review and develops our hypothesis. Section 3 describes the sample and research design. Section 4 presents our main empirical results. Section 5 concludes the paper.

## Literature review and hypothesis development

### Literature review of political turnover

The influence of institutional environment and political turnover on both macro and micro economies has long captured the attention of scholars [17, 18].

From a macro perspective, a plethora of global research highlights that political uncertainty hampers effective fiscal redistribution by governments, undermining the pursuit of egalitarianism [1]. In the Chinese context, local officials prioritize economic growth as a crucial performance indicator, and political turnover serves as a significant mechanism through which the government can shape economic outcomes via personnel control [2]. However, political turnover has adverse consequences, as it leads local officials to prioritize economic benefits over environmental concerns such as air quality and pollution control, thus impeding the achievement of environmental goals [8, 9].

From a micro perspective, the relationship between the government and companies plays a pivotal role in shaping firms’ operations and profitability. Political turnover introduces significant external uncertainty for firms [19], influencing their decision-making processes [3], strategic changes [4], and investment efficiency [5]. It also impacts firms’ charitable donations and acts as a deterrent to corporate corruption [20]. Julio and Yook (2012) using cross-country data from 48 nations, found that political uncertainty surrounding national elections reduces firms’ investment spending [21]. This effect is also observed in China’s local political turnover. The change of provincial secretaries can hinder companies’ investment [6], particularly when new officials hail from different provinces [10]. Using the Economic Policy Uncertainty Index as a measure of political uncertainty, Díaz-Díaz et al. (2022) discovered that political uncertainty reduces Spanish firms’ investment in research and development (R&D) and innovation [22]. Conversely, researchers in China have shown that uncertainty caused by local political turnover has a positive impact on firms’ R&D investment [7], incentivizing their innovative behavior and number of patent applications [23]. Additionally, political turnover negatively impacts companies’ speculative behavior, such as investing in financial products and securities [24]. For foreign companies, domestic political turnover can affect their willingness to enter China, particularly when new leaders hail from different cities or change abnormally [12]. Political uncertainty also impacts financial markets. In China, changes in local Communist Party secretaries increase the risk of stock price collapse in local firms, particularly for non-state-owned companies and those with less transparency [25]. Political turnover also raises the possibility of bond defaults [26] and increases the cost of equity financing [27].

However, existing literature primarily examines the impact of political turnover on the economy, corporate investment, and finance. Given that the Chinese government is now emphasizing the environment, social welfare, and corporate compliance, we will further focus on the influence of political turnover on corporate environmental, social, and governance (ESG) performance.

### Hypothesis development

Political turnover introduces significant external uncertainty for companies, leading them to adopt strategies to manage this uncertainty, such as reducing the quantity and quality of information disclosure. In this context, environmental, social responsibility, and corporate governance (ESG) have emerged as crucial dimensions of corporate rating standards and investment philosophies that prioritize sustainability over traditional financial performance. Rating agencies assess a company’s ESG performance by evaluating the information disclosed by the company on environmental practices, social responsibility initiatives, and corporate governance policies. These evaluations contribute to the construction of an ESG performance indicator. This study aims to investigate the impact of political turnover on corporate ESG performance in China. The analysis is conducted by examining three key aspects.

Firstly, the government’s performance evaluation heavily relies on Gross Domestic Product (GDP) as the core indicator, which creates an incentive for local governments to prioritize "visible" economic and financial performance over "invisible" sustainable performance, such as environmental and social responsibility. Local officials are evaluated based on their ability to drive economic growth, as better economic performance enhances their chances of promotion [28]. As a result, new officials following political turnover are under pressure to demonstrate their capacity and dedication to improving economic performance through policy implementation and reforms during their tenure. Listed companies play a pivotal role in promoting economic growth and are valuable resources for regional economic development. Consequently, local officials are likely to prioritize the financial performance of listed companies over their ESG performance to enhance their personal prospects for advancement. However, this focus on short-term economic gains and disregard for ESG performance can lead to myopic policies. Furthermore, the change in government officials introduces greater external political uncertainty for listed companies, distorts their investment behavior, and reduces investment efficiency [29].

Secondly, unlike in developed countries, local officials in China are appointed by the central government rather than being elected by local voters. This difference in the political system and government transitions creates a lack of transparency and unpredictability for the general public. Following a change in officials, enterprises may face challenges in predicting the behavior, preferences, and priorities of the new officials [10]. Consequently, they may adopt a more cautious approach, reducing the level of strategic change [4] and dedicating more time to enhancing their financial performance and cultivating a positive relationship with the newly appointed officials [30–32]. This shift in focus towards short-term objectives and relationship-building may come at the expense of long-term sustainability goals, resulting in a decline in ESG performance.

Based on the aforementioned points, it is evident that enterprises may exhibit under-investment behavior in response to political turnover. They may prioritize maintaining a favorable relationship with the government and meeting the performance assessment criteria set by government officials, which could result in the diversion of capital and human resources away from long-term development initiatives [8]. Consequently, this lack of funding support may impede the implementation of ESG-related projects within these enterprises.

Thirdly, it is important to note that investment in environmental protection, social responsibility, and corporate governance typically does not yield immediate financial returns for companies. Therefore, the improvement of a company’s ESG performance often requires government policies and subsidies to provide guidance and support. Government subsidies and policy initiatives play a crucial role in enhancing corporate ESG performance and encouraging sustainable investments [33–35]. However, within the political system in China, there exists a common issue known as "new officials ignoring former commitments." This means that newly appointed officials may not acknowledge the contracts, commitments, debts, and actions made by their predecessors. Consequently, there is a risk of discontinuity in local government policies, as new officials may prioritize their own performance targets and show reluctance to support the initiatives and policies of their predecessors. As a result, the support policies and subsidies previously provided by former officials for corporate ESG development may be delayed or denied. This lack of support and motivation can discourage enterprises from undertaking sustainable development projects, ultimately leading to a decline in their ESG performance.

Combining the above factors, this study proposes the hypothesis:

**Hypothesis: Political turnover will reduce corporate ESG performance.**

## Research design

### Sample and data

To conduct the empirical analysis, our sample included all the firm-year observations of Chinese A-share-listed companies from 2010 to 2020. We selected 2010 as the starting year of the sample because it is the year when ESG score data provided by Huazheng became available. To ensure the sample’s reliability, we excluded listed companies within the financial industry, companies that are delisted or labeled as ST/*ST, and observations with missing variables. The ESG scores were obtained from the Huazheng ESG rating, while information on political turnover was collected from the Chinese Research Data Service Platform (CBRDS). Other relevant data were sourced from the China Stock Market and Accounting Research Database (CSMAR). In total, our final sample comprised 30,627 firm-year observations. To mitigate the impact of extreme values, we applied winsorization to the continuous variables at the 1% and 99% levels.

Table 1 reports the distribution of the samples. On average, 24.1% (25.5%) of the samples belong to cities where the municipal party committee secretary (the mayor) changed that year. This proportion reached its highest point in 2016, amounting to 35.9% (35.0%).

**Table 1:** Distribution of turnovers of local officials during 2010–2020.

Year	Number of samples	Mayor turnover (%)	Party sectary turnover (%)
2010	1843	27.3%	19.0%
2011	2149	31.9%	30.4%
2012	2350	22.0%	15.2%
2013	2333	32.3%	21.0%
2014	2358	13.7%	13.9%
2015	2539	28.6%	27.8%
2016	2758	35.0%	35.9%
2017	3197	31.2%	32.7%
2018	3473	27.8%	30.6%
2019	3624	22.0%	24.0%
2020	4003	14.1%	13.0%
Total	30627	25.5%	24.1%

### Research methodology

To analyze the relationship between political turnover and corporate ESG performance, the research model is designed as follows:

$${\text{ESG}}_{i,t}=\beta_{0}+\beta_{1}{\text{PT}}_{i,t}+{\text{Controls}}_{i,t}+\text{Firm}+\text{Year}+\varepsilon_{i,t}$$ (1)

Our main attention is on the statistical significance and economic meaning of the estimation of *β*_*1*_. If *β*_*1*_ is significantly negative, it means that political turnover has a negative impact on corporate ESG performance, and a positive otherwise.

According to previous studies [36–38], the dependent variable *ESG* is measured by Huazheng ESG. Based on the core connotation and development experience of ESG, combined with the actual situation of the domestic market, the Huazheng ESG evaluation system constructs a three-level index system from top to bottom. Specifically, there are 3 first-level indicators, 14 second-level indicators, and 26 third-level indicators, with more than 130 underlying data indicators. Through quarterly regular evaluations and dynamic tracking, the ESG score levels of all A-share listed companies have been systematically measured over the past 10 years, on a scale of 0 to 100 points. These scores are then assigned corresponding ratings from "AAA" to "C", representing high to low performance. Therefore, we assign values from 1 to 9 for the variable ESG, corresponding to the ratings from "C" to "AAA".

The independent variable is *PT*, which represents political turnover. We distinguish two different types of political turnover: external appointments of officials from higher-level government and local promotions. Given that local officials hold significant power in formulating economic policies within their jurisdictions, and due to variations in personal experiences and governing philosophies, there is substantial heterogeneity in each official’s approach to policy formulation, leading to distinct regional economic policies. Therefore, we focus only on cases of external appointments when considering *PT*. Following previous studies [39, 40], we used the turnover of the municipal party committee secretary or the mayor of the prefecture-level city where the firm is located as our measure of political turnover. The party heads are the local party committee secretaries, namely the city party secretary. The government heads are the local chief executives, namely the mayor. A local government is headed by the local party secretary and the mayor, who act as the first and second leaders, respectively [2]. *PT* is a dummy variable equal to 1 if the mayor or the party secretary in the firm i’s city changes in year t, and 0 otherwise. Moreover, *Mayor* is a dummy variable equal to 1 if the mayor in the firm i’s city changes in year t, and 0 otherwise. *Psecretary* is a dummy variable equal to 1 if the party secretary in the firm i’s city changes in year t, and 0 otherwise.

Following existing literature in corporate finance [41, 42], we control other variables influencing ESG performance, including firm size (*Size*), asset-liability ratio (*Lev*), return on asset (*ROA*), revenue growth rate (*Growth*), board independence (*Indep*), market performance (*TobinQ*), CEO duality (*Duality*), ownership concentration (*Top10*), firm age (*Age*), local economic condition (*Per capita GDP*). We also control firm-fixed effects and year-fixed effects in our model to reduce estimation bias. Appendix A in S1 Table provides a detailed definition of all the variables.

### Descriptive statistics

Table 2 shows the descriptive statistics of the main variables in the regression test. The mean of *PT*, *Mayor*, and *Psecretary* is 0.360, 0.255, and 0.241, suggesting that 36% of the observations are confronted with prefecture-level city political turnover, to be specific, 25.5% are faced with mayor turnover and 24.1% are faced with municipal party committee secretary turnover. On average, 43.1% of the observations’ assets are financed by liabilities, and companies have an 18% growth rate in revenue each year.

**Table 2:** Descriptive statistics.

VARIABLES	N	Mean	SD	Min	P25	P50	P75	Max
ESG	30627	6.455	1.130	1.000	6.000	6.000	7.000	9.000
PT	30627	0.360	0.480	0.000	0.000	0.000	1.000	1.000
Mayor	30627	0.255	0.436	0.000	0.000	0.000	1.000	1.000
Psecretary	30627	0.241	0.428	0.000	0.000	0.000	0.000	1.000
Size	30627	22.140	1.356	19.420	21.180	21.950	22.890	26.370
Lev	30627	0.431	0.218	0.051	0.256	0.420	0.591	0.980
ROA	30627	0.040	0.070	-0.297	0.014	0.039	0.073	0.232
Growth	30627	0.180	0.482	-0.642	-0.029	0.105	0.268	3.324
Indep	30627	0.375	0.054	0.300	0.333	0.357	0.429	0.571
TobinQ	30627	2.298	1.658	0.897	1.328	1.758	2.599	10.940
Duality	30627	0.277	0.448	0.000	0.000	0.000	1.000	1.000
Top10	30627	0.359	0.223	0.009	0.170	0.349	0.534	0.855
Age	30627	9.014	0.241	8.311	8.876	9.035	9.223	9.510
Per capita GDP	30627	9.632	4.005	0.602	6.689	9.385	13.060	21.550

*No*te. This table reports the descriptive statistics of the main variables for samples from 2010–2020. See Appendix A in S1 Table for the definition of all variables.

## Empirical results

### Baseline results

We estimate equation (1) with *PT*, *Mayor*, and *Psecretary* as independent variables to measure political turnover. The results are presented in Table 3. In column (1), we regress PT on ESG without including control variables, while in column (2), we include all the control variables in the regression. As anticipated by our theoretical analysis, the estimations of *β*_*1*_ are significantly negative at the 1% level in both column (1) and column (2). These findings indicate that political turnover has a significantly negative impact on corporate ESG performance.

**Table 3:** Baseline results.

VARIABLES	(1)	(2)	(3)	(4)
	ESG	ESG	ESG	ESG
PT	-0.0740***	-0.0519***		
	(-3.691)	(-2.797)		
Mayor			-0.0549***	
			(-3.178)	
Psecretary				-0.0316*
				(-1.783)
Size		0.5521**	0.5526**	0.5548**
		(2.044)	(2.040)	(2.045)
Lev		0.0874	0.0871	0.0882
		(1.302)	(1.297)	(1.313)
ROA		3.3668***	3.3660***	3.3704***
		(21.389)	(21.377)	(21.419)
Growth		-0.0712***	-0.0709***	-0.0709***
		(-5.518)	(-5.494)	(-5.499)
Indep		0.0923	0.0908	0.0968
		(0.444)	(0.437)	(0.466)
TobinQ		-0.0992***	-0.0993***	-0.0994***
		(-14.816)	(-14.822)	(-14.815)
Duality		0.0929***	0.0927***	0.0928***
		(4.635)	(4.627)	(4.631)
Top10		0.8616***	0.8626***	0.8634***
		(16.294)	(16.299)	(16.302)
Age		0.0811	0.0813	0.0813
		(1.461)	(1.464)	(1.463)
Per capita GDP		0.0083**	0.0083**	0.0084**
		(2.315)	(2.300)	(2.329)
Constant	5.8246***	4.7084***	4.7053***	4.6933***
	(23.530)	(8.399)	(8.393)	(8.369)
Year & Firm	Yes	Yes	Yes	Yes
Observations	30627	30627	30627	30627
R2	0.111	0.192	0.192	0.191
Adj-R2	0.108	0.189	0.189	0.189

*Note*. This table reports the baseline results from OLS regression, with the first column without control variables and the other columns with all the variables. The dependent variable is *ESG*, and the independent variables in column (2), column (3), and column (4) are *PT*, *Mayor*, and *Psecretary*. Firm-fixed effects and year-fixed effects are also included. The t values reported in parentheses are adjusted based on robust standard errors clustered by firm, where *, **, and *** denote significance levels of 0.01, 0.05, and 0.10 respectively.

These findings indicate that political turnover has a significantly negative impact on corporate ESG performance. Specifically, when controlling for all the control variables in column (2), the estimation of *β*_*1*_ is -0.0519. This suggests that, on average, companies in cities experiencing political turnover have their ESG score reduced by 0.0519 compared to those without political turnover. ESG scoring is a comprehensive system, although the final result of ESG is only reflected in a specific number or even a small number, but the factors reflected behind it are comprehensive. The interpret the magnitude of the coefficients are statistically significant, and the economic significance lies in quarterly regular evaluations and dynamic tracking, the impact on ESG can be seen quickly and consistently. These results support our hypothesis that political turnover diminishes corporate ESG performance. Regarding the control variables, our analysis indicates that companies located in regions with higher economic performance benefit from greater resources and policy support for investing in ESG. *Per capita GDP* demonstrates a positive correlation with ESG, which is significant at the 5% level.

To further analyze the distinct roles of mayor and party committee secretary, we conduct regressions of *Mayor* on *ESG* in column (3) and *Psecretary* on *ESG* in column (4) respectively. The absolute value of *β*_*1*_ in column (3) is 0.0549, which is larger than that in column (4), and the results in column (3) demonstrate stronger statistical significance. This suggests that, on average, companies in cities experiencing mayor turnover have their ESG score reduced more than companies in cities experiencing secretary turnover. The discrepancy between *Mayor* and *Psecretary* can be attributed to the political structure and division of responsibilities in China. Mayors are entrusted with overseeing overall municipal development, including local economic growth, investment attraction, social welfare, and environmental protection. On the other hand, party committee secretaries primarily focus on Party affairs, strengthening Party leadership, and overseeing the appointment and dismissal of Party members. They also collaborate with the mayor to accomplish economic development tasks [2, 6]. Consequently, mayors exert a more substantial influence on ESG performance.

### Mechanism analysis

#### Governmental subsidies

Since governmental subsidies and support have been found to significantly affect corporate sustainable development [33, 35], political turnover introduces uncertainty and inconsistency to subsidy policies. Therefore, we hypothesize that the uncertainty arising from political turnover reduces government subsidies, which diminishes companies’ incentive to invest in ESG, ultimately resulting in reduced ESG performance. To quantify the variable *Subsidy*, we examine the disclosure of governmental subsidies in the Notes to Financial Statements of listed companies. To test the mediating effect, we employ Baron and Kenny’s (1986) method [43]. The results in Table 4 shed light on the underlying mechanisms. In column (1), the coefficient of *PT* is -0.1550, significant at the 1% level, indicating that political turnover negatively impacts the receipt of governmental subsidies by companies. Column (3) reveals a positive association between governmental subsidies and corporate ESG performance, and shows that the estimated coefficient of the influence of *PT* on *ESG* is negative and statistically significant. Furthermore, column (5) reveals a positive association between PT*Subsidy and *ESG* at the 1% level. However, when compared with the results in Table 2, column (2), the absolute value of the estimation is lower but still significant at the 5% level. Thus, governmental subsidies demonstrate a partial mediating effect, suggesting that political turnover leads to a decrease in governmental subsidies, consequently exerting a negative impact on corporate ESG performance.

**Table 4:** Merchanism analysis.

VARIABLES	(1)	(2)	(3)	(4)	(5)	(6)
	Subsidy	UnderInvest	ESG	ESG	ESG	ESG
PT	-1.1550***	0.0010***	-0.0470**	-0.0502***	-0.0667***	-0.0674***
	(-3.370)	(2.737)	(-2.549)	(-2.707)	(-3.388)	(-3.489)
Subsidy			0.0040***		0.0035**	
			(2.664)		(2.345)	
UnderInvest				-1.6296***		-2.1455***
				(-5.940)		(-5.880)
PT* Subsidy					0.0052***	
					(2.879)	
PT*UnderInvest						-1.1307**
						(-2.443)
Size	67.8685*	-0.0019	0.2779	0.5492**	0.2962	0.5464**
	(1.726)	(-1.438)	(1.586)	(2.044)	(1.585)	(2.049)
Lev	9.2110***	-0.0096***	0.0493	0.0715	0.0337	0.0687
	(4.123)	(-8.858)	(0.713)	(1.068)	(0.499)	(1.026)
ROA	17.6456***	-0.0468***	3.2952***	3.2901***	3.2793***	3.3015***
	(4.756)	(-13.879)	(20.699)	(20.854)	(20.909)	(20.944)
Growth	-0.3066	0.0017***	-0.0699***	-0.0686***	-0.0700***	-0.0683***
	(-1.324)	(4.795)	(-5.429)	(-5.323)	(-5.429)	(-5.305)
Indep	14.1203**	0.0097***	0.0407	0.1082	0.0245	0.0948
	(2.543)	(2.976)	(0.198)	(0.522)	(0.120)	(0.459)
TobinQ	-0.7475***	0.0017***	-0.0962***	-0.0965***	-0.0954***	-0.0965***
	(-5.082)	(11.064)	(-14.303)	(-14.454)	(-14.313)	(-14.427)
Duality	-0.0083	0.0015***	0.0930***	0.0955***	-0.1052***	-0.1120***
	(-0.021)	(4.649)	(4.696)	(4.760)	(-4.651)	(-4.873)
Top10	7.5634***	0.0189***	0.8327***	0.8913***	0.8253***	0.8866***
	(4.162)	(23.306)	(15.984)	(16.660)	(15.928)	(16.582)
Age	-0.1314	0.0050***	0.0815	0.0894	0.0725	0.0861
	(-0.104)	(6.643)	(1.493)	(1.610)	(1.328)	(1.549)
Per capita GDP	0.1880***	-0.0000	0.0075**	0.0083**	0.0077**	0.0086**
	(3.010)	(-0.429)	(2.111)	(2.313)	(2.170)	(2.392)
Constant	-8.3555	-0.0420***	4.7411***	4.6393***	5.0211***	4.8748***
	(-0.801)	(-5.940)	(8.568)	(8.261)	(9.107)	(8.681)
Year & Firm	Yes	Yes	Yes	Yes	Yes	Yes
Observations	30627	30627	30627	30627	30627	30627
R2	0.295	0.082	0.198	0.193	0.199	0.193
Adj-R2	0.293	0.0786	0.195	0.190	0.196	0.191

*Note*. This table reports the mechanisms of the relationship between political turnover and corporate ESG performance. Column (1) and column (3) test the mechanism by governmental subsidies while column (2) and column (4) test the mechanism by under-investment. The t values reported in parentheses are adjusted based on robust standard errors clustered by firm, where *, **, and *** denote significance levels of 0.01, 0.05, and 0.10 respectively. All variables are defined in Appendix A in S1 Table.

There are two plausible explanations for this phenomenon. From the perspective of enterprises, political turnover introduces uncertainty to the policy environment, leading to a reduction in government subsidies. Faced with such uncertainty, companies tend to decrease their investment in ESG initiatives and hold more cash, adopting a precautionary approach to navigate the political uncertainty associated with political turnover. From the government’s standpoint, during periods of political turnover, there is increased uncertainty in the local policy environment, coupled with strong central supervision. Local officials tend to maintain some flexibility in their economic behavior to navigate this uncertainty, resulting in a decrease in governmental subsidies. Additionally, driven by incentives for political promotion, competition, and fiscal growth, new officials prioritize financial performance indicators, such as fiscal revenue and tax revenue, over ESG performance.

#### Under-investment

Political factors can have a distorting effect on companies’ investment efficiency and increase external political uncertainty, ultimately leading to a neglect of long-term ESG investment. We hypothesize that the pressure exerted by new officials to prioritize GDP assessments reduces companies’ incentives to invest in ESG initiatives, resulting in under-investment and a subsequent decrease in ESG performance. Following the research conducted by Richardson (2006) [44], we utilized the GMM method to estimate the components of investment, namely expected investment and abnormal investment (the residual error in the equation). The negative value of abnormal investment indicates under-investment, and thus we define the variable *UnderInvest* as the absolute value of the negative estimated residual error, otherwise set it to 0. The results concerning the mediation effect of under-investment are presented in Table 4. In column (2), the coefficient of *PT* is significantly positive, indicating that political turnover leads to corporate under-investment. In column (4), both the coefficients of *UnderInvest* and *PT* are significantly negative at the level of 1%, suggesting that under-investment serves as one of the mechanisms linking political turnover and corporate ESG performance. Furthermore, column (6) reveals a negative association between PT*UnderInvest and *ESG* at the 5% level. These findings imply that political turnover results in companies under-investing, thereby diminishing their corporate ESG performance. In other words, under-investment plays a partial intermediary role in the relationship between political turnover and corporate ESG performance.

One possible explanation for this phenomenon can be understood through the lens of real options theory. ESG investment, in comparison to other types of investments, is often irreversible and entails a higher risk of failure. Moreover, the benefits derived from ESG investments typically require a significant amount of time to materialize. In the context of political turnover and the associated uncertainty surrounding industry policies and regulations, enterprises tend to prioritize immediate gains and are inclined to reduce their commitments to ESG investment. This preference for short-term interests can lead to a decrease in ESG investment.

### Robustness tests

#### Alternative measure of the dependent variable

Even though we add *Per capita GDP* as a control variable to control the influence of local economic growth, the baseline regression cannot avoid estimation bias like individual differences. To control individual differences between companies, we use the variable *diffESG* to measure the change in corporate ESG performance. The variable *diffESG* is defined as the difference between ESG score in year t and year t-1. The results are shown in Table 5, column (1) shows the influence of political turnover (including mayor and secretary), column (2) shows the influence of mayor turnover, and column (3) shows that of secretary turnover. The coefficient of *PT* is significantly negative at the 5% level, which means that political turnover has a negative impact on corporate ESG performance. The impact also exists in the relationship between mayor turnover and corporate ESG performance, as is shown in column (2). However, in column (3), the coefficient of *Psecretary* has neither statistical significance nor economic significance. The results confirm our hypothesis that political turnover has a negative impact on corporate ESG performance.

**Table 5:** Alternative measure of dependent variable.

VARIABLES	(1)	(2)	(3)
	diffESG	diffESG	diffESG
PT	-0.0180**		
	(-1.978)		
Mayor		-0.0192*	
		(-1.820)	
Psecretary			-0.0034
			(-0.328)
Size	0.0616**	0.0618**	0.0628**
	(2.573)	(2.555)	(2.563)
Lev	-0.0159	-0.0161	-0.0156
	(-0.810)	(-0.817)	(-0.794)
ROA	1.2161***	1.2159***	1.2175***
	(15.158)	(15.156)	(15.176)
Growth	0.0051	0.0052	0.0052
	(0.489)	(0.500)	(0.502)
Indep	-0.0504	-0.0508	-0.0483
	(-0.781)	(-0.786)	(-0.748)
TobinQ	-0.0040*	-0.0041*	-0.0041*
	(-1.692)	(-1.702)	(-1.721)
Duality	0.0184**	0.0184**	0.0184**
	(2.288)	(2.280)	(2.282)
Top10	0.0536***	0.0539***	0.0545***
	(2.858)	(2.876)	(2.905)
Age	0.0154	0.0155	0.0155
	(1.103)	(1.110)	(1.107)
Per capita GDP	-0.0002	-0.0002	-0.0001
	(-0.218)	(-0.245)	(-0.176)
Constant	-0.2484*	-0.2500*	-0.2551*
	(-1.841)	(-1.853)	(-1.889)
Year & Firm	Yes	Yes	Yes
Observations	30627	30627	30627
R2	0.042	0.042	0.042
Adj-R2	0.038	0.038	0.038

*Note*. This table reports the robustness tests from OLS regression. The dependent variable is *diffESG*, and the independent variables in column (1), column (2), and column (3) are *PT*, *Mayor*, and *Psecretary* respectively. Firm-fixed effects and year-fixed effects are also included. The t values reported in parentheses are adjusted based on robust standard errors clustered by firm, where *, **, and *** denote significance levels of 0.01, 0.05, and 0.10 respectively. All variables are defined in Appendix A in S1 Table.

#### Measurement the dependent variable based on other data provider

Apart from Huazheng, there are other data providers that also publish ESG rating data for Chinese firms. In this section, we employed Hexun ESG rating data as an alternative measure for the dependent variable ESG in order to assess the consistency of our regression results. We utilized the variable HXESG to capture corporate ESG performance based on the Hexun ESG rating data. The findings, presented in Table 6, demonstrate that column (1) to column (3) exhibit regression relationships between *HXESG* and *PT*, *Mayor*, and *Secretary*, respectively. Notably, the signs and significance of the coefficients align with the baseline results, further reinforcing the robustness of our findings.

**Table 6:** Measurement the dependent variable based on other data provider.

VARIABLES	(1)	(2)	(3)
	HXESG	HXESG	HXESG
PT	-0.1405***		
	(-2.763)		
Mayor		-0.2577**	
		(-2.325)	
Psecretary			-0.0452*
			(-1.740)
Size	3.4196***	3.4156***	3.4279***
	(2.622)	(2.620)	(2.621)
Lev	0.2840	0.2779	0.2878
	(0.510)	(0.500)	(0.517)
ROA	96.8812***	96.8637***	96.8938***
	(68.708)	(68.690)	(68.734)
Growth	0.0195	0.0197	0.0192
	(0.124)	(0.125)	(0.122)
Indep	-0.9606	-0.9771	-0.9444
	(-0.578)	(-0.588)	(-0.568)
TobinQ	-0.9812***	-0.9807***	-0.9819***
	(-14.786)	(-14.789)	(-14.790)
Duality	-0.1996	-0.1985	-0.2005
	(-1.051)	(-1.046)	(-1.056)
Top10	5.3069***	5.3083***	5.3110***
	(12.055)	(12.057)	(12.057)
Age	0.3030	0.3062	0.3009
	(0.768)	(0.776)	(0.763)
Per capita GDP	0.1282***	0.1274***	0.1281***
	(5.052)	(5.031)	(5.052)
Constant	11.2273***	11.2009***	11.2219***
	(2.824)	(2.819)	(2.824)
Year & Firm	Yes	Yes	Yes
Observations	21,751	21,751	21,751
R2	0.404	0.404	0.404
Adj-R2	0.401	0.401	0.401

*Note*. This table reports the tests of the measurement the dependent variable based on other data provider. Firm-fixed effects and year-fixed effects are also included. The t values reported in parentheses are adjusted based on robust standard errors clustered by firm, where *, **, and *** denote significance levels of 0.01, 0.05, and 0.10 respectively. All variables are defined in Appendix A in S1 Table.

#### Individual measure of the ESG

In this section, we conducted regression tests on each aspect of ESG, namely the environmental score, social score, and governance score, individually. The results are presented in Table 7, with column (1) to column (3) displaying the regression relationships between political turnover and the environmental score, social score, and governance score, respectively. Similarly, column (4) to column (6) showcase the regression relationships between mayor turnover and each aspect of ESG, while column (7) to column (9) exhibit the regression relationships between secretary turnover and each aspect of ESG. The results intuitively reveal that political turnover has a more pronounced direct effect on the environmental score and social score, as opposed to the governance score. The possible explanation behind this phenomenon lies in China’s special political system, if core government officials, such as mayors and party secretaries, are changed, the economic policies, regional development policies and environmental protection policies pursued by former officials during their tenure are all at risk of change. In particular, the relatively stable relationship between government and enterprise and the government and business ecology may be “reshuffled” before the change of core officials. These changes are profoundly affecting how companies respond, with environmental, social and governance implications; However, the environmental and social performance reflects the external response behaviour of enterprises, which is the most direct impact on the former economic policies, regional development policies, environmental protection policies and other external changes. The external response behaviours of enterprises are more easily observed by the market and investors. In order to avoid the external risk of policy uncertainty, enterprises will adopt a more conservative strategy. The performance of governance is the internal response behaviour of enterprises, which is less visible to the market and investors, and the degree of external influence is relatively small.

**Table 7:** Individual measure of the ESG.

VARIABLES	(1)	(2)	(3)	(4)	(5)	(6)	(7)	(8)	(9)
	E	S	G	E	S	G	E	S	G
PT	-0.3190**	-0.3214**	-0.2986***						
	(-2.447)	(-2.056)	(-2.983)						
Mayor				-0.3087**	-0.3578**	-0.2636***			
				(-2.555)	(-2.385)	(-2.707)			
Psecretary							-0.2271*	-0.2931*	-0.2047*
							(-1.684)	(-1.901)	(-1.814)
Size	2.7037***	3.8656***	2.3736**	2.6979***	3.8532***	2.3804**	2.6945***	3.8575***	2.3864**
	(2.589)	(3.118)	(2.021)	(2.598)	(3.130)	(2.017)	(2.587)	(3.116)	(2.022)
Lev	3.1938***	2.2251***	-8.5168***	3.1950***	2.2230***	-8.5172***	3.1904***	2.2221***	-8.5124***
	(6.744)	(3.852)	(-20.920)	(6.748)	(3.847)	(-20.909)	(6.737)	(3.847)	(-20.893)
ROA	9.7492***	22.2052***	25.0296***	9.7515***	22.1966***	25.0297***	9.7291***	22.1855***	25.0503***
	(8.708)	(16.512)	(24.128)	(8.711)	(16.501)	(24.120)	(8.692)	(16.506)	(24.154)
Growth	-0.8099***	-0.3104**	-0.5646***	-0.8115***	-0.3122**	-0.5631***	-0.8110***	-0.3113**	-0.5632***
	(-9.428)	(-2.266)	(-5.615)	(-9.448)	(-2.279)	(-5.598)	(-9.433)	(-2.273)	(-5.602)
Indep	-1.6955	-2.7226	18.2542***	-1.6922	-2.7369	18.2549***	-1.7135	-2.7386	18.2787***
	(-1.168)	(-1.602)	(16.295)	(-1.166)	(-1.610)	(16.284)	(-1.180)	(-1.611)	(16.301)
TobinQ	-0.8195***	-0.7115***	-0.5860***	-0.8193***	-0.7109***	-0.5863***	-0.8192***	-0.7112***	-0.5866***
	(-16.550)	(-10.771)	(-12.135)	(-16.545)	(-10.762)	(-12.134)	(-16.541)	(-10.770)	(-12.132)
Duality	-0.5835***	0.1505	-0.4715***	-0.5841***	0.1506	-0.4712***	-0.5837***	0.1502	-0.4717***
	(-3.307)	(0.762)	(-3.619)	(-3.310)	(0.762)	(-3.616)	(-3.306)	(0.760)	(-3.620)
Top10	2.6162***	1.4229***	1.6075***	2.6094***	1.4125***	1.6146***	2.6095***	1.4170***	1.6166***
	(7.296)	(3.336)	(5.424)	(7.282)	(3.315)	(5.442)	(7.281)	(3.325)	(5.448)
Age	-0.3194	-2.6769***	-0.8930***	-0.3204	-2.6775***	-0.8922***	-0.3207	-2.6783***	-0.8922***
	(-0.807)	(-5.980)	(-2.983)	(-0.810)	(-5.979)	(-2.978)	(-0.811)	(-5.982)	(-2.978)
Per capita GDP	0.0184	0.0122	0.0799***	0.0187	0.0123	0.0797***	0.0180	0.0118	0.0803***
	(0.722)	(0.414)	(4.128)	(0.734)	(0.417)	(4.116)	(0.706)	(0.401)	(4.142)
Constant	66.3466***	81.9536***	90.6284***	66.3762***	82.0165***	90.5936***	66.4244***	82.0285***	90.5436***
	(18.143)	(19.472)	(27.833)	(18.147)	(19.481)	(27.788)	(18.159)	(19.486)	(27.767)
Year & Firm	Yes	Yes	Yes	Yes	Yes	Yes	Yes	Yes	Yes
Observations	30627	30627	30627	30627	30627	30627	30627	30627	30627
R^2^	0.193	0.264	0.263	0.193	0.264	0.263	0.193	0.264	0.263
Adj-R^2^	0.191	0.261	0.260	0.190	0.261	0.260	0.191	0.261	0.260

*Note*. This table reports the tests of Individual measure of the ESG. Firm-fixed effects and year-fixed effects are also included. The t values reported in parentheses are adjusted based on robust standard errors clustered by firm, where *, **, and *** denote significance levels of 0.01, 0.05, and 0.10 respectively.

#### Controlling the provincial political turnover

In this section, considering provincial political turnover may affect both municipal political turnover and corporate ESG performance, we need to further control the provincial political turnover. So, we introduce new variables *PPT* (dummy variable equals 1 if the provincial governor or party secretary of the company i changed in year t), *PMayor* (dummy variable equals 1 if the provincial governor of the company i changed in year t), and *PPsecretary* (dummy variable equals 1 if provincial party secretary of the company i changed in year t) to control the provincial political turnover separately. The results are shown in Table 8, after controlling provincial political turnover the estimations of the coefficient of *PT* are still negative, with strong statically and economic significance. In short, our hypothesis still holds after considering provincial turnover.

**Table 8:** Controlling the provincial political turnover.

VARIABLES	(1)	(2)	(3)
	ESG	ESG	ESG
PT	-0.0553***	-0.0534***	-0.0522***
	(-3.090)	(-2.946)	(-2.865)
PPT	-0.0215*		
	(-1.713)		
PMayor		-0.0222*	
		(-1.784)	
PPsecretary			-0.0033
			(-0.204)
Size	0.5523**	0.5523**	0.5521**
	(2.041)	(2.043)	(2.044)
Lev	0.0875	0.0875	0.0874
	(1.304)	(1.304)	(1.302)
ROA	3.3656***	3.3661***	3.3668***
	(21.381)	(21.383)	(21.387)
Growth	-0.0712***	-0.0712***	-0.0712***
	(-5.514)	(-5.515)	(-5.518)
Indep	0.0912	0.0921	0.0922
	(0.439)	(0.443)	(0.444)
TobinQ	-0.0992***	-0.0992***	-0.0992***
	(-14.808)	(-14.812)	(-14.814)
Duality	0.0931***	0.0930***	0.0929***
	(4.644)	(4.640)	(4.636)
Top10	0.8618***	0.8617***	0.8616***
	(16.294)	(16.294)	(16.294)
Age	0.0814	0.0813	0.0811
	(1.467)	(1.465)	(1.462)
Per capita GDP	0.0084**	0.0084**	0.0083**
	(2.336)	(2.333)	(2.316)
Constant	4.7014***	4.7040***	4.7077***
	(8.383)	(8.388)	(8.397)
Year & Firm	Yes	Yes	Yes
Observations	30627	30627	30627
R2	0.192	0.192	0.192
Adj-R2	0.189	0.189	0.189

*Note*. This table reports the robustness tests from OLS regression with provincial political turnover fixed. The dependent variable is *ESG*. Firm-fixed effects and year-fixed effects are also included. The t values reported in parentheses are adjusted based on robust standard errors clustered by firm, where *, **, and *** denote significance levels of 0.01, 0.05, and 0.10 respectively. All variables are defined in Appendix A in S1 Table.

#### Instrumental variable test

Refer to An et al. (2016) [10], we employed an instrumental variable (IV) approach to mitigate potential endogeneity concerns in our revised manuscript. We selected three instrumental variables: tenure, age, and education level of local government officials. These variables are known to influence government official changes but are not directly associated with ESG performance, indicating the strength of our IVs. In the first stage, we regress PT, Mayor, Psecretary on the three IVs and control variables used in Table 2. Columns 1–3 of Table 9 report the first stage regression results. All three IVs are significantly related to political turnover. Specifically, the mayor and party secretary are more likely to be replaced if they are younger, have longer tenures, and have higher levels of education. In the second stage, we replace PT, Mayor, Psecretary by its predicated value from the first-stage regression. The results reported in columns 4–6 of Table 9 suggest that the negative relationship between political turnover and ESG performance continues to hold after correcting for any potential endogeneity bias. Moreover, we conduct the under-identification test and weak-identification test, the results of the under-identification test are all significant at 1%, which shows the IVs are significantly correlated with endogenous variables. The results of the under-recognition test are all higher than 10, which proves that there is no weak instrumental variable problem, so the instrumental variable is effective.

**Table 9:** Instrumental variable test.

VARIABLES	Frist Stage			Second Stage		
	(1)	(2)	(3)	(4)	(5)	(6)
	PT	Mayor	Psecretary	ESG_HZ	ESG_HZ	ESG_HZ
PT				-0.0211***		
				(-3.017)		
Mayor					-0.0162***	
					(-3.004)	
Psecretary						-0.0115*
						(-1.784)
Tenure	0.2975***	0.2011***	0.2004***			
	(3.003)	(3.016)	(3.056)			
Age	-0.2020***	-0.1988***	-0.2013***			
	(-3.039)	(-3.096)	(-3.126)			
Education	0.2014***	0.2003***	0.2592***			
	(3.007)	(3.206)	(2.927)			
asset	-0.0773	-0.0633**	-0.0399	0.5537**	0.5537**	0.5537**
	(-1.529)	(-2.044)	(-1.015)	(2.045)	(2.045)	(2.046)
Lev	-0.0250	-0.0291	-0.0171	0.0863	0.0863	0.0864
	(-1.114)	(-1.625)	(-0.997)	(1.285)	(1.285)	(1.286)
ROA	-0.0570	-0.0722*	-0.0154	3.3834***	3.3834***	3.3834***
	(-1.141)	(-1.718)	(-0.369)	(21.505)	(21.505)	(21.509)
Growth	-0.0060	0.0002	-0.0031	-0.0704***	-0.0704***	-0.0704***
	(-1.099)	(0.040)	(-0.667)	(-5.462)	(-5.460)	(-5.462)
Indirector	-0.1206*	-0.1313**	-0.0752	0.0846	0.0846	0.0850
	(-1.729)	(-2.332)	(-1.388)	(0.408)	(0.408)	(0.410)
TobinQ	0.0025	0.0023	0.0005	-0.0995***	-0.0995***	-0.0995***
	(1.083)	(1.196)	(0.276)	(-14.798)	(-14.797)	(-14.792)
Duality	0.0017	0.0037	0.0031	-0.1092***	-0.1092***	-0.1092***
	(0.206)	(0.546)	(0.470)	(-4.756)	(-4.756)	(-4.755)
Top10	-0.0424**	-0.0257*	-0.0218	0.8584***	0.8584***	0.8583***
	(-2.442)	(-1.840)	(-1.625)	(16.193)	(16.193)	(16.190)
Age	0.0030	0.0036	0.0064	0.0766	0.0766	0.0767
	(0.157)	(0.241)	(0.456)	(1.378)	(1.378)	(1.380)
GDP_PerCapita	-0.0010	-0.0023***	0.0002	0.0086**	0.0086**	0.0086**
	(-0.950)	(-2.613)	(0.245)	(2.393)	(2.393)	(2.395)
Constant	0.0452	0.0447	-0.0161	4.9215***	4.9213***	4.9249***
	(0.243)	(0.308)	(-0.119)	(8.776)	(8.776)	(8.782)
Year & Firm	Yes	Yes	Yes	Yes	Yes	Yes
Observations	30,627	30,627	30,627	30,627	30,627	30,627
R^2^	0.161	0.122	0.138	0.191	0.191	0.192
Adj-R^2^	0.158	0.120	0.135	0.189	0.189	0.189
Underidentification test				4.626***	5.015***	4.218***
Weak identification test				14.601	14.311	12.108

*Note*. This table reports the results of instrumental variable test. Firm-fixed effects and year-fixed effects are also included. The t values reported in parentheses are adjusted based on robust standard errors clustered by firm, where *, **, and *** denote significance levels of 0.01, 0.05, and 0.10 respectively.

#### Placebo test

In line with previous research conducted by Dong et al. (2022) [5], we conducted a counterfactual placebo test to validate the robustness of our results and examine the specific influence of political turnover on corporate ESG performance, as distinct from other factors. The purpose of this test was to assess whether artificial scenarios of officials’ turnover would have any real impact on corporate ESG performance if the change of officials genuinely influenced it. We constructed a "counterfactual" situation by artificially advancing the timing of political turnover by 1 year, resulting in three new variables: *LPT* (a dummy variable equal to 1 if the municipal party committee secretary or mayor of the prefecture-level city of company i changed in year t-1), *LMayor* (a dummy variable equal to 1 if the mayor of the prefecture-level city of company i changed in year t-1), and *LPsecretary* (a dummy variable equal to 1 if the municipal party committee secretary of company i changed in year t-1). The regression results, displayed in Table 10, reveal that the estimated coefficients of *LPT*, *LMayor*, and *LPsecretary* remain significantly negative, albeit with a slightly lower level of significance. These findings align with the observations made by Dong et al. (2022) [5] and may be attributed to the possibility of leaked information about political turnover, resulting in the anticipated effects of turnover being realized even before the actual change takes place.

**Table 10:** Placebo test.

VARIABLES	(1)	(2)	(3)
	ESG	ESG	ESG
LPT	-0.0363**		
	(-1.985)		
LMayor		-0.0366**	
		(-2.137)	
LPsecretary			-0.0123*
			(-1.707)
Size	0.4958**	0.4962**	0.4972**
	(1.986)	(1.983)	(1.989)
Lev	0.1233*	0.1230*	0.1236*
	(1.865)	(1.861)	(1.869)
ROA	2.3395***	2.3393***	2.3418***
	(15.239)	(15.237)	(15.261)
Growth	-0.0867***	-0.0865***	-0.0865***
	(-6.369)	(-6.352)	(-6.355)
Indep	0.1879	0.1875	0.1909
	(0.896)	(0.894)	(0.910)
TobinQ	-0.0936***	-0.0936***	-0.0936***
	(-13.902)	(-13.906)	(-13.904)
Duality	0.0830***	0.0828***	0.0830***
	(4.141)	(4.135)	(4.142)
Top10	0.7556***	0.7563***	0.7567***
	(13.966)	(13.968)	(13.975)
Age	0.0845	0.0848	0.0847
	(1.479)	(1.483)	(1.482)
Per capita GDP	0.0089**	0.0088**	0.0089**
	(2.457)	(2.447)	(2.472)
Constant	4.7406***	4.7369***	4.7308***
	(8.310)	(8.303)	(8.290)
Year & Firm	Yes	Yes	Yes
Observations	27,555	27,555	27,555
R2	0.177	0.177	0.177
Adj-R2	0.174	0.174	0.174

*Notes*. This table reports the placebo test from OLS regression. The dependent variable is *ESG*, and the independent variables in column (1), column (2) and column (3) are *LPT*, *LMayor*, and *LPsecretary* respectively. Firm-fixed effects and year-fixed effects are also included. The t values reported in parentheses are adjusted based on robust standard errors clustered by firm, where *, **, and *** denote significance levels of 0.01, 0.05, and 0.10 respectively. All variables are defined in Appendix A in S1 Table.

Then, we deliberate “moving up” the time of political turnover by 2 years and generate 3 new variables: *L2PT* (dummy variable equals 1 if the municipal party committee secretary or the mayor of the prefecture-level city of the company i changed in year t-2), *L2Mayor* (dummy variable equals 1 if the mayor of the prefecture-level city of the company i changed in year t-2), and *L2Psecretary* (dummy variable equals 1 if he municipal party committee secretary of the company i changed in year t-2). The results of the regression are shown in Table 11, column (1) is the results of the regression of *L2PT* to ESG, column (2) is the results of the regression of *L2Mayor* to ESG, and column (3) is the results of the regression of *L2Psecretary* to ESG. All the estimated coefficients are not significantly different from 0 statically. The results indicate that the impact of political turnover is not random and our results are robust.

**Table 11:** Placebo test.

VARIABLES	(1)	(2)	(3)
	ESG	ESG	ESG
L2PT	-0.0178		
	(-0.977)		
L2Mayor		-0.0232	
		(-1.340)	
L2Psecretary			-0.0226
			(-1.306)
Size	0.2918***	0.2919***	0.2919***
	(3.493)	(3.498)	(3.498)
Lev	-0.5011***	-0.5015***	-0.5012***
	(-7.604)	(-7.608)	(-7.606)
ROA	0.4266***	0.4257***	0.4274***
	(3.067)	(3.060)	(3.074)
Growth	-0.0947***	-0.0947***	-0.0946***
	(-6.972)	(-6.967)	(-6.961)
Indep	0.0077	0.0062	0.0091
	(0.038)	(0.031)	(0.045)
TobinQ	-0.0049	-0.0049	-0.0049
	(-0.693)	(-0.691)	(-0.694)
Duality	0.0307	0.0306	0.0307
	(1.614)	(1.610)	(1.614)
Top10	0.3672***	0.3674***	0.3673***
	(6.526)	(6.529)	(6.527)
Age	0.0953*	0.0955*	0.0954*
	(1.704)	(1.707)	(1.705)
Per capita GDP	0.0061*	0.0061*	0.0062*
	(1.760)	(1.743)	(1.774)
Constant	-1.2001*	-1.2016*	-1.2039*
	(-1.933)	(-1.936)	(-1.939)
Year & Firm	Yes	Yes	Yes
Observations	23,391	23,391	23,391
R2	0.230	0.230	0.230
Adj-R2	0.226	0.226	0.226

*Note*. This table reports the results of the placebo test from OLS regression. The dependent variable is *ESG*, and the independent variables in column (1), column (2) and column (3) are *L2PT*, *L2Mayor*, and *L2Psecretary* respectively. Firm-fixed effects and year-fixed effects are also included. The t values reported in parentheses are adjusted based on robust standard errors clustered by firm, where *, **, and *** denote significance levels of 0.01, 0.05, and 0.10 respectively. All variables are defined in Appendix A in S1 Table.

### Across-sectional tests

#### State-owned enterprise

In the context of China, state-owned enterprises (SOEs) are mandated to fulfill their social responsibilities as government-controlled shareholders, thereby setting an example for other enterprises. Apart from achieving financial performance targets, SOEs are expected to share in social responsibilities encompassing the environment, livelihood, and employment. Consequently, SOEs exhibit greater emphasis on ESG investment and demonstrate better ESG performance compared to non-SOEs [39]. We hypothesized that due to the divergent actual controllers and interest orientations of SOEs and non-SOEs, the impact of government officials’ turnover would be less pronounced in SOEs.

To examine the differential effects of political turnover on corporate ESG performance between SOEs and non-SOEs, we introduced the variable *State*. Specifically, *State* is assigned a value of 1 if the company is a state-owned enterprise and 0 if it is a non-state-owned enterprise. The results, presented in Table 12, reveal significant and positive relationships between the interaction of *PT* and *State*, as well as *Mayor* and *State*, with *ESG*. These findings indicate that the influence of political turnover on SOEs’ ESG performance is comparatively diminished in comparison to non-SOEs, thus supporting our initial assumption.

**Table 12:** Moderating effect of ownership.

VARIABLES	(1)	(2)	(3)
	ESG	ESG	ESG
PT	-0.0358*		
	(-1.861)		
Mayor		-0.0384**	
		(-2.134)	
Psecretary			-0.0220
			(-1.192)
PT*State	0.0903*		
	(1.659)		
Mayor*State		0.1104**	
		(2.053)	
Psecretary*State			0.0395
			(0.749)
State	0.3415***	0.3385***	0.3217***
	(7.206)	(7.419)	(7.102)
Size	0.5606**	0.5610**	0.5626**
	(2.004)	(2.001)	(2.004)
Lev	0.0482	0.0474	0.0486
	(0.727)	(0.715)	(0.733)
ROA	3.3200***	3.3188***	3.3241***
	(21.132)	(21.119)	(21.161)
Growth	-0.0687***	-0.0683***	-0.0686***
	(-5.336)	(-5.300)	(-5.323)
Indep	0.1587	0.1583	0.1634
	(0.771)	(0.769)	(0.793)
TobinQ	-0.0967***	-0.0967***	-0.0969***
	(-14.480)	(-14.488)	(-14.500)
Duality	0.0807***	0.0807***	0.0805***
	(3.991)	(3.992)	(3.985)
Top10	0.7972***	0.7977***	0.7992***
	(15.002)	(15.010)	(15.021)
Age	0.0661	0.0663	0.0660
	(1.198)	(1.201)	(1.196)
Per capita GDP	0.0079**	0.0078**	0.0080**
	(2.206)	(2.196)	(2.250)
Constant	4.8081***	4.8061***	4.7971***
	(8.630)	(8.626)	(8.614)
Year & Firm	Yes	Yes	Yes
Observations	30627	30627	30627
R2	0.198	0.198	0.197
Adj-R2	0.195	0.195	0.194

*Note*. This table reports the OLS regression results of the moderating effect of ownership on the relationship between political turnover and corporate ESG performance. We use *State* to measure whether the company is an SOE. PT*State, Mayor*State, and Psecretary*State are multiplication of *PT* and *State*, *Mayor*, and *State*, and *Psecretaery* and *State* respectively. Firm-fixed effects and year-fixed effects are also included. The t values reported in parentheses are adjusted based on robust standard errors clustered by firm, where *, **, and *** denote significance levels of 0.01, 0.05, and 0.10 respectively. All variables are defined in Appendix A in S1 Table.

#### Political connection

Political connections provide firms with access to valuable policy information. When managers have networks and ties with government officials, they gain greater access to information and can mitigate uncertainty [45, 46]. Politically connected firms are better positioned to obtain information about how the incoming officials will alter existing policies, introduce new policies, and interpret policy preferences, states, and trends. By leveraging their political connections, these firms experience fewer disruptions from government officials’ turnover and can sustain their investments in ESG. We hypothesize that politically connected firms will experience a reduced negative impact of political turnover on their corporate ESG performance. As a result, they are better equipped to navigate the challenges arising from changes in government officials and maintain their commitment to sustainable ESG investments.

In this section, we use the dummy variable *Policon* to carry out an analysis of whether the political connection can influence the relationship between political turnover and corporate ESG performance. *Policon* equals 1 if any of the chairmen of the board and CEO of the enterprise is or was a government official, and 0 otherwise. Table 13 shows the results of political connection’s moderating effect. The coefficients of PT*Policon, Mayor*Policon, and Psecretary*Policon are significantly positive. The results support our assumption that political connection with governmental officials can mitigate the negative impact of political turnover on corporate ESG performance.

**Table 13:** Moderating effect of political connection.

VARIABLES	(1)	(2)	(3)
	ESG	ESG	ESG
PT	-0.0670***		
	(-3.312)		
Mayor		-0.0712***	
		(-3.750)	
Psecretary			-0.0013
			(-1.629)
PT*Policon	0.0663*		
	(1.835)		
Mayor*Policon		0.0695*	
		(1.939)	
Psecretary*Policon			0.0875**
			(2.326)
Policon	0.0104	0.0166	0.0126
	(0.342)	(0.572)	(0.443)
Size	0.5496**	0.5498**	0.5529**
	(2.043)	(2.038)	(2.047)
Lev	0.0909	0.0903	0.0911
	(1.356)	(1.346)	(1.358)
ROA	3.3664***	3.3657***	3.3691***
	(21.393)	(21.380)	(21.425)
Growth	-0.0718***	-0.0714***	-0.0716***
	(-5.564)	(-5.533)	(-5.550)
Indep	0.0879	0.0869	0.0936
	(0.424)	(0.419)	(0.452)
TobinQ	-0.0988***	-0.0988***	-0.0990***
	(-14.722)	(-14.725)	(-14.730)
Duality	0.0930***	0.0928***	0.0929***
	(4.646)	(4.638)	(4.641)
Top10	0.8624***	0.8633***	0.8637***
	(16.317)	(16.322)	(16.319)
Age	0.0834	0.0837	0.0832
	(1.505)	(1.510)	(1.501)
Per capita GDP	0.0085**	0.0085**	0.0086**
	(2.378)	(2.364)	(2.382)
Constant	4.6839***	4.6789***	4.6720***
	(8.356)	(8.349)	(8.334)
Year & Firm	Yes	Yes	Yes
Observations	30627	30627	30627
R2	0.192	0.192	0.192
Adj-R2	0.189	0.189	0.189

*Note*. This table reports the OLS regression results of the moderating effect of ownership on the relationship between political turnover and corporate ESG performance. We use *Policon* to measure whether the company has a strong political connection with the government, if any of the chairman and general manager of the enterprise is or was a government official, *Policon* equals 1, and 0 otherwise. PT*Policon, Mayor*Policon, and Psecretary*Policon are multiplication of *PT* and *Policon*, *Mayor* and *Policon*, and *Psecretaery* and *Policon* respectively. Firm-fixed effects and year-fixed effects are also included. The t values reported in parentheses are adjusted based on robust standard errors clustered by firm, where *, **, and *** denote significance levels of 0.01, 0.05, and 0.10 respectively. All variables are defined in Appendix A in S1 Table.

#### Industrial structure

The industrial structure plays a crucial role in economic growth and sustainable development [47, 48]. Typically, regions with well-developed tertiary industries have a competitive edge in economic development and possess ample technical and financial resources to prioritize ESG investments. On the other hand, enterprises operating in the primary and secondary industries, such as agriculture and manufacturing, often face challenges in meeting financial performance targets while dealing with high levels of pollutant emissions, inadequate working conditions, and insufficient safety measures, resulting in environmental and social responsibility issues. As a consequence, these industries require substantial investments in ESG practices, which can lead to lower overall ESG performance. We hypothesize that the negative impact of political turnover on corporate ESG performance is less pronounced in the tertiary industries. This is because regions with well-developed tertiary sectors tend to have a more robust foundation for sustainable development and are better equipped to address ESG concerns.

We define the variable *GDP3* to indicate the industrial structure if the proportion of the tertiary industry GDP in the total GDP in the region where the listed company is located is higher than the annual median, which means that the tertiary industry is relatively developed, so *GDP3* equals 1, 0 otherwise. The results are shown in Table 14, the coefficients of PT*GDP3, Mayor*GDP3, and Psecretary*GDP3 are significantly positive, which suggests that highly developed tertiary industry can mitigate the negative impact of political turnover on corporate ESG performance, supporting our assumption.

**Table 14:** Moderating effect of industrial structure.

VARIABLES	(1)	(2)	(3)
	ESG	ESG	ESG
PT	-0.0030		
	(-0.144)		
Mayor		-0.0006	
		(-0.028)	
Psecretary			-0.0151
			(-0.744)
PT*GDP3	0.0735**		
	(2.410)		
Mayor*GDP3		0.0812***	
		(2.699)	
Psecretary*GDP3			0.0682**
			(2.215)
GDP3	0.1042***	0.0974***	0.0991***
	(3.873)	(3.804)	(3.899)
Size	0.5432**	0.5441**	0.5449**
	(2.037)	(2.033)	(2.036)
Lev	0.0887	0.0870	0.0885
	(1.323)	(1.298)	(1.320)
ROA	3.3732***	3.3717***	3.3748***
	(21.431)	(21.412)	(21.447)
Growth	-0.0716***	-0.0717***	-0.0711***
	(-5.546)	(-5.557)	(-5.509)
Indep	0.0778	0.0778	0.0792
	(0.376)	(0.376)	(0.383)
TobinQ	-0.0990***	-0.0991***	-0.0991***
	(-14.828)	(-14.840)	(-14.837)
Duality	0.0924***	0.0924***	0.0920***
	(4.615)	(4.612)	(4.597)
Top10	0.8581***	0.8594***	0.8589***
	(16.286)	(16.296)	(16.287)
Age	0.0811	0.0811	0.0811
	(1.466)	(1.464)	(1.465)
Per capita GDP	0.0057	0.0056	0.0058
	(1.594)	(1.553)	(1.630)
Constant	4.6626***	4.6676***	4.6608***
	(8.343)	(8.352)	(8.341)
Year & Firm	Yes	Yes	Yes
Observations	30627	30627	30627
R2	0.193	0.193	0.193
Adj-R2	0.190	0.190	0.190

*Note*. This table reports the OLS regression results of the moderating effect of ownership on the relationship between political turnover and corporate ESG performance. We use *GDP3* to measure industrial structure, if the proportion of the tertiary industry GDP in the total GDP in the region where the listed company is located is higher than the annual median, *GDP3* equals 1, otherwise equals 0. PT*GDP3, Mayor*GDP3, and Psecretary*GDP3 are the multiplication of *PT* and *GDP3*, *Mayor* and *GDP3*, and *Psecretaery* and *GDP3* respectively. Firm-fixed effects and year-fixed effects are also included. The t values reported in parentheses are adjusted based on robust standard errors clustered by firm, where *, **, and *** denote significance levels of 0.01, 0.05, and 0.10 respectively. All variables are defined in Appendix A in S1 Table.

## Conclusion

This study aims to examine the impact of political turnover on corporate ESG performance in China. By controlling for various factors that previous literature has identified as influential on corporate ESG performance, we find that the turnover of the municipal party committee secretary or the mayor of the prefecture-level city where a firm is located leads to a reduction in corporate ESG performance. Specifically, we observe that the change of mayor has a more pronounced negative effect on ESG performance compared to the change of the party committee. To better understand the mechanisms underlying this phenomenon, we investigate the transmission channels involved. Our analysis reveals that political turnover, due to the resulting policy uncertainty, leads to a decrease in governmental subsidies and encourages companies to engage in ineffective under-investment. Consequently, this diminishes corporate ESG performance. Additionally, a series of robustness checks reinforce our findings, indicating that potential endogeneity is unlikely to drive the observed relationship.

Our research findings have important practical implications. Firstly, they highlight the significance of political uncertainty resulting from political turnover as a significant barrier to achieving ESG performance goals. As a result, managers must carefully assess the potential for political turnover in their region when formulating ESG investment strategies. Secondly, policymakers should strive to comprehend the economic mechanisms underlying the negative association between political turnover and corporate ESG performance. This understanding can aid new officials in guiding local resource allocation towards environmental and social responsibility effectively. Furthermore, our study offers empirical evidence emphasizing the importance of state-owned enterprises (SOEs), political connections, and tertiary industries in managing political uncertainty. In environments with heightened political uncertainty, listed companies need to strike a balance between political connections and resource utilization to gain a competitive advantage. Overall, our study contributes valuable insights that can inspire regulators in emerging markets to establish appropriate mechanisms that mitigate the negative impact of political uncertainty stemming from changes in government officials.

## Supporting information

S1 Table Appendix A. (DOCX)

## References

[1] Vu T.V., 2022. Unbundling the effect of political instability on income redistribution. Eur. J. Polit. Econ. 75, 102189. doi: 10.1016/j.ejpoleco.2022.102189

[2] Li H., Zhou L.-A., 2005. Political turnover and economic performance: the incentive role of personnel control in China. J. Public Econ. 89, 1743–1762. doi: 10.1016/j.jpubeco.2004.06.009

[3] Gonçalves T., Barros V., Serra G., 2022. Political elections uncertainty and earnings management: Does firm size really matter? Econ. Lett. 214, 110438. doi: 10.1016/j.econlet.2022.110438

[4] Choi S., Liu H., Yin J., Qi Y., Lee J.Y., 2021. The effect of political turnover on firms’ strategic change in the emerging economies: The moderating role of political connections and financial resources. J. Bus. Res. 137, 255–266. doi: 10.1016/j.jbusres.2021.08.034

[5] Dong Z., Wang X., Zhang T., Zhong Y., 2022. The effects of local government leadership turnover on entrepreneurial behavior. China Econ. Rev. 71, 101727. doi: 10.1016/j.chieco.2021.101727

[6] Chen Y., 2022. Does political turnover affect corporate investment? Evidence from China. Emerg. Mark. Rev. 51, 100865. doi: 10.1016/j.ememar.2021.100865

[7] Cao Y., Chen Y., Zhang Y., 2022. Political uncertainty, innovation-driven strategy, and corporate R&D. Res. Int. Bus. Finance 60, 101612. doi: 10.1016/j.ribaf.2021.101612

[8] Deng Y., Wu Y., Xu H., 2019. Political turnover and firm pollution discharges: An empirical study. China Econ. Rev. 58, 101363. doi: 10.1016/j.chieco.2019.101363

[9] Song C., Sesmero J., Delgado M.S., 2021. The effect of uncertain political turnover on air quality: Evidence from China. J. Clean. Prod. 303, 127048. doi: 10.1016/j.jclepro.2021.127048

[10] An H., Chen Y., Luo D., Zhang T., 2016. Political uncertainty and corporate investment: Evidence from China. J. Corp. Finance 36, 174–189. doi: 10.1016/j.jcorpfin.2015.11.003

[11] Chen Y., Chen D., Wang W., Zheng D., 2018. Political uncertainty and firms’ information environment: Evidence from China. J. Account. Public Policy 37, 39–64. doi: 10.1016/j.jaccpubpol.2018.01.005

[12] Chen S., Mao H., Feng Z., 2020. Political uncertainty and firm entry: Evidence from Chinese manufacturing industries. J. Bus. Res. 120, 16–30. doi: 10.1016/j.jbusres.2020.07.021

[13] Broadstock D.C., Chan K., Cheng L.T.W., Wang X., 2021. The role of ESG performance during times of financial crisis: Evidence from COVID-19 in China. Finance Res. Lett. 38, 101716. doi: 10.1016/j.frl.2020.101716 32837385 PMC7425769

[14] Fatemi A., Glaum M., Kaiser S., 2018. ESG performance and firm value: The moderating role of disclosure. Glob. Finance J. 38, 45–64. doi: 10.1016/j.gfj.2017.03.001

[15] Ng A.C., Rezaee Z., 2015. Business sustainability performance and cost of equity capital. J. Corp. Finance.

[16] Wang Z., Sarkis J., 2017. Corporate social responsibility governance, outcomes, and financial performance. J. Clean. Prod. 162, 1607–1616. doi: 10.1016/j.jclepro.2017.06.142

[17] La Porta R., Lopez‐de‐Silanes F., Shleifer A., Vishny R.W., 1998. Law and Finance. J. Polit. Econ. 106, 1113–1155. doi: 10.1086/250042

[18] Shleifer A., Vishny R., 1994. Politicians and Firms. Q. J. Econ. 109, 995–1025. doi: 10.2307/2118354

[19] Luo D., Chen K.C., Wu L., 2017. Political uncertainty and firm risk in China. Rev. Dev. Finance 7, 85–94. doi: 10.1016/j.rdf.2017.06.001

[20] Cheng M., Geng H., 2021. Do local firms employ political activities to respond to political uncertainty? J. Asian Econ. 73, 101270. doi: 10.1016/j.asieco.2020.101270

[21] Julio B., Yook Y., 2012. Political Uncertainty and Corporate Investment Cycles. J. Finance 67, 45–83. doi: 10.1111/j.1540-6261.2011.01707.x

[22] Díaz-Díaz N.L., López-Iturriaga F.J., Santana-Martín D.J., 2022. The role of political ties and political uncertainty in corporate innovation. Long Range Plann. 55, 102111. doi: 10.1016/j.lrp.2021.102111

[23] Chen Y., 2021. Does political turnover stifle or stimulate corporate innovation? Int. Rev. Econ. Finance 76, 1126–1145. doi: 10.1016/j.iref.2021.08.010

[24] Aibai A., Peng Y., Shen P., Xu H., 2022. Can local policy uncertainty curtail corporate speculation on financial assets? Int. Rev. Financ. Anal. 83, 102287. doi: 10.1016/j.irfa.2022.102287

[25] Yu J., Mai D., 2020. Political turnover and stock crash risk: Evidence from China. Pac.-Basin Finance J. 61, 101324. doi: 10.1016/j.pacfin.2020.101324

[26] Zou J., Li S., Xu Z., Deng G., 2022. Political uncertainty and bond defaults: evidence from the Chinese market. Eur. J. Finance. doi: 10.1080/1351847X.2022.2089048

[27] Li X., Luo J., Chan K.C., 2018. Political uncertainty and the cost of equity capital. Finance Res. Lett. 26, 215–222. doi: 10.1016/j.frl.2018.01.009

[28] Chen J., Luo D., She G., Ying Q., 2017. Incentive or Selection? A New Investigation of Local Leaders’ Political Turnover in China. Soc. Sci. Q. 98, 341–359. doi: 10.1111/ssqu.12280

[29] Chen S., Sun Z., Tang S., Wu D., 2011. Government intervention and investment efficiency: Evidence from China. J. Corp. Finance 17, 259–271. doi: 10.1016/j.jcorpfin.2010.08.004

[30] Jia M., Zhang Z., 2018. The Role of Corporate Donations in Chinese Political Markets. J. Bus. Ethics 153, 519–545. doi: 10.1007/s10551-016-3378-1

[31] Kamidi A., Guo J., 2022. The impact of political turnover on corporate misconduct and philanthropy: evidence from China. Asian Bus. Manag. doi: 10.1057/s41291-022-00210-5

[32] Liu W., De Sisto M., Li W.H., 2021. How does the turnover of local officials make firms more charitable? A comprehensive analysis of corporate philanthropy in China. Emerg. Mark. Rev. 46, 100748. doi: 10.1016/j.ememar.2020.100748

[33] Chen, Dimitrov S., Pun H., 2019. The impact of government subsidy on supply Chains’ sustainability innovation. Omega-Int. J. Manag. Sci. 86, 42–58. doi: 10.1016/j.omega.2018.06.012

[34] Huang Z., Liao G., Li Z., 2019. Loaning scale and government subsidy for promoting green innovation. Technol. Forecast. Soc. Change 144, 148–156. doi: 10.1016/j.techfore.2019.04.023

[35] Yang X., He L., Xia Y., Chen Y., 2019. Effect of government subsidies on renewable energy investments: The threshold effect. Energy Policy 132, 156–166. doi: 10.1016/j.enpol.2019.05.039

[36] Chen Z., Xie G., 2022. ESG disclosure and financial performance: Moderating role of ESG investors. Int. Rev. Financ. Anal. 83, 102291. doi: 10.1016/j.irfa.2022.102291

[37] Jiang Y., Wang C., Li S., Wan J., 2022. Do institutional investors’ corporate site visits improve ESG performance? Evidence from China. Pac.-Basin Finance J. 76, 101884. doi: 10.1016/j.pacfin.2022.101884

[38] Deng X., Li W., Ren X., 2023. More sustainable, more productive: Evidence from ESG ratings and total factor productivity among listed Chinese firms. Finance Res. Lett. 51, 103439. doi: 10.1016/j.frl.2022.103439

[39] Huang J., 2022. Corporate social responsibility and financial performance: The moderating role of the turnover of local officials. Finance Res. Lett. 46, 102497. doi: 10.1016/j.frl.2021.102497

[40] Li M., Cao Y., Lu M., Wang H., 2021. Political uncertainty and allocation of decision rights among business groups: Evidence from the replacement of municipal officials. Pac.-Basin Finance J. 67, 101541. doi: 10.1016/j.pacfin.2021.101541

[41] Bolourian S., Angus A., Alinaghian L., 2021. The impact of corporate governance on corporate social responsibility at the board-level: A critical assessment. J. Clean. Prod. 291, 125752. doi: 10.1016/j.jclepro.2020.125752

[42] Ge W., Ouyang C., Shi Z., Chen Z., 2022. Can a not-for-profit minority institutional shareholder make a big difference in corporate governance? A quasi-natural experiment. J. Corp. Finance 72, 102125. doi: 10.1016/j.jcorpfin.2021.102125

[43] Baron R., Kenny D., 1986. The Moderator Mediator Variable Distinction in Social Psychological-Research—Conceptual, Strategic, and Statistical Considerations. J. Pers. Soc. Psychol. 51, 1173–1182. doi: 10.1037//0022-3514.51.6.1173 3806354

[44] Richardson S., 2006. Over-investment of free cash flow. Rev. Account. Stud. 11, 159–189. doi: 10.1007/s11142-006-9012-1

[45] Krammer S.M.S., Jiménez A., 2020. Do political connections matter for firm innovation? Evidence from emerging markets in Central Asia and Eastern Europe. Technol. Forecast. Soc. Change 151, 119669. doi: 10.1016/j.techfore.2019.05.027

[46] Wang Z., Fu H., Ren X., 2023. Political connections and corporate carbon emission: New evidence from Chinese industrial firms. Technol. Forecast. Soc. Change 188, 122326. doi: 10.1016/j.techfore.2023.122326

[47] Hao X., Li Y., Ren S., Wu H., Hao Y., 2023. The role of digitalization on green economic growth: Does industrial structure optimization and green innovation matter? J. Environ. Manage. 325, 116504. doi: 10.1016/j.jenvman.2022.116504 36272290

[48] Song M., Tao W., Shen Z., 2022. Improving high-quality development with environmental regulation and industrial structure in China. J. Clean. Prod. 366, 132997. doi: 10.1016/j.jclepro.2022.132997

