# Supplementary material for: The effect of political turnover on corporate ESG performance: Evidence from China
Source: PLoS One. 2023 Jul 24;18(7):e0288789. doi: 10.1371/journal.pone.0288789 (PMC10365318; doi:10.1371/journal.pone.0288789)
Supplement: S1 Table — (DOCX) [file pone.0288789.s001.docx]

**Supporting Information**

**Table. Appendix A**

| Variable name | Definition |
| --- | --- |
| *ESG* | Huazheng ESG rating |
| *PT* | A dummy variable equals 1 if the municipal party committee secretary or the mayor of the prefecture-level city of the company i changed in year t, and 0 otherwise |
| *Mayor* | A dummy variable equals 1 if the mayor of the prefecture-level city of the company i changed in year t, and 0 otherwise |
| *Psecretary* | A dummy variable equals 1 if the municipal party committee secretary of the company i, changed in year t, and 0 otherwise |
| *Size* | Natural logarithm of the total assets. |
| *Lev* | The ratio of total debt to total assets. |
| *ROA* | The ratio of operating profit to total assets |
| *Growth* | Revenue growth rate |
| *Indep* | The ratio of independent directors to the board of directors |
| *TobinQ* | (Net assets per share × number of non-tradable shares + price per share × number of tradable shares + book value of liabilities) / Total assets |
| *Duality* | A dummy variable equals 1 if a firm’s CEO is also the chair of the board, and 0 otherwise |
| *Top10* | The sum of squares of the shareholding ratio of top ten shareholders |
| *Age* | Ln (year – listing year +1) |
| *Per capita GDP* | Per capita GDP of the city where the firm located in year t |
| *Subsidy* | The number of governmental subsidies |
| *UnderInvest* | Based on the research of Richardson (2006), taking the absolute value of negative estimated residual error, otherwise, equals 0 |
| *diffESG* | The difference between ESG rating in year t and year t-1 |
| *PPT* | A dummy variable equals 1 if the provincial governor or party secretary of the company i changed in year t, and 0 otherwise |
| *PMayor* | A dummy variable equals 1 if the provincial governor of the company i changed in year t, and 0 otherwise |
| *PPsecretary* | A dummy variable equals 1 if the provincial party secretary of the company i changed in year t, and 0 otherwise |
| *LPT* | A dummy variable equals 1 if the municipal party committee secretary or the mayor of the prefecture-level city of the company i changed in year t-1, and 0 otherwise |
| *LMayor* | A dummy variable equals 1 if the mayor of the prefecture-level city of the company i changed in year t-1, and 0 otherwise |
| *LPsecretary* | A dummy variable equals 1 if the municipal party committee secretary of the company i changed in year t-1, and 0 otherwise |
| *L2PT* | A dummy variable equals 1 if the municipal party committee secretary or the mayor of the prefecture-level city of the company i changed in year t-2, and 0 otherwise |
| *L2Mayor* | A dummy variable equals 1 if the mayor of the prefecture-level city of the company i changed in year t-2, and 0 otherwise |
| *L2Psecretary* | A dummy variable equals 1 if the municipal party committee secretary of the company i changed in year t-2, and 0 otherwise |
| *State* | A dummy variable equals 1 if the company is an SOE, and 0 otherwise |
| *Policon* | A dummy variable equals 1 if any of the chairman and general manager of the enterprise is or was a government official, and 0 otherwise |
| *GDP3* | A dummy variable equals 1 if proportion of the tertiary industry GDP in the total GDP in the region where the listed company is located is higher than the annual median, and 0 otherwise |
